# Study and implementation of urogenital schistosomiasis elimination in Zanzibar (Unguja and Pemba islands) using an integrated multidisciplinary approach

**DOI:** 10.1186/1471-2458-12-930

**Published:** 2012-10-30

**Authors:** Stefanie Knopp, Khalfan A Mohammed, Said M Ali, I Simba Khamis, Shaali M Ame, Marco Albonico, Anouk Gouvras, Alan Fenwick, Lorenzo Savioli, Daniel G Colley, Jürg Utzinger, Bobbie Person, David Rollinson

**Affiliations:** 1 Department of Epidemiology and Public Health, Swiss Tropical and Public Health Institute, P.O. Box,, CH–4002, Basel, Switzerland; 2 University of Basel, P.O. Box,, CH–4003, Basel, Switzerland; 3 Wolfson Wellcome Biomedical Laboratories, Department of Life Sciences, Natural History Museum, Cromwell Road, London, SW7 5BD, UK; 4 Helminth Control Laboratory Unguja, Ministry of Health, P.O. Box 236, Zanzibar, United Republic of Tanzania; 5 Public Health Laboratory - Ivo de Carneri, Ministry of Health, P.O. Box 122, Chake-Chake, Pemba, United Republic of Tanzania; 6 Ivo de Carneri Foundation, Viale Monza 44, 20127, Milan, Italy; 7 Schistosomiasis Control Initiative, Department of Infectious Disease Epidemiology, Faculty of Medicine, VB1 Norfolk Place, St. Mary's Campus, London, UK; 8 Department of Control of Neglected Tropical Diseases, World Health Organization, 20 Avenue Appia, CH-1211, Geneva 27, Switzerland; 9 Department of Microbiology and Center for Tropical and Emerging Global Diseases, University of Georgia, 330B Coverdell Building, Athens, GA, 30602, USA; 10 National Center for Preparedness, Detection, and Control of Infectious Diseases, Centers for Disease Control and Prevention, 1600 Clifton Road, Mailstop C-14, Atlanta, GA, 30333, USA

**Keywords:** Urogenital schistosomiasis, *Schistosoma haematobium*, Preventive chemotherapy, Snail control intervention, *Bulinus globosus*, Behaviour change intervention, Morbidity control, Transmission control, Elimination, Zanzibar, Tanzania

## Abstract

**Background:**

Schistosomiasis is a parasitic infection that continues to be a major public health problem in many developing countries being responsible for an estimated burden of at least 1.4 million disability-adjusted life years (DALYs) in Africa alone. Importantly, morbidity due to schistosomiasis has been greatly reduced in some parts of the world, including Zanzibar. The Zanzibar government is now committed to eliminate urogenital schistosomiasis. Over the next 3–5 years, the whole at-risk population will be administered praziquantel (40 mg/kg) biannually. Additionally, snail control and behaviour change interventions will be implemented in selected communities and the outcomes and impact measured in a randomized intervention trial.

**Methods/Design:**

In this 5-year research study, on both Unguja and Pemba islands, urogenital schistosomiasis will be assessed in 45 communities with urine filtration and reagent strips in 4,500 schoolchildren aged 9–12 years annually, and in 4,500 first-year schoolchildren and 2,250 adults in years 1 and 5. Additionally, from first-year schoolchildren, a finger-prick blood sample will be collected and examined for *Schistosoma haematobium* infection biomarkers. Changes in prevalence and infection intensity will be assessed annually. Among the 45 communities, 15 were randomized for biannual snail control with niclosamide, in concordance with preventive chemotherapy campaigns. The reduction of *Bulinus globosus* snail populations and *S. haematobium*-infected snails will be investigated. In 15 other communities, interventions triggering behaviour change have been designed and will be implemented in collaboration with the community. A change in knowledge, attitudes and practices will be assessed annually through focus group discussions and in-depth interviews with schoolchildren, teachers, parents and community leaders. In all 45 communities, changes in the health system, water and sanitation infrastructure will be annually tracked by standardized questionnaire-interviews with community leaders. Additional issues potentially impacting on study outcomes and all incurring costs will be recordedand monitored longitudinally.

**Discussion:**

Elimination of schistosomiasis has become a priority on the agenda of the Zanzibar government and the international community. Our study will contribute to identifying what, in addition to preventive chemotherapy, needs to be done to prevent, control, and ultimately eliminate schistosomiasis, and to draw lessons for current and future schistosomiasis elimination programmes in Africa and elsewhere.

**Trial registration:**

ISRCTN48837681

## Background

### Burden and transmission of schistosomiasis, with focus on *Schistosoma haematobium* infection in Zanzibar

Schistosomiasis is a group of diseases caused by parasitic worms of the genus *Schistosoma*. These blood-dwelling flukes have a complicated life cycle involving freshwater snail intermediate hosts and transmission of the parasite is governed by social-ecological systems and intimately linked with conditions of poverty [1,2]. More than 200 million people are infected with more than 95% of the infections concentrated in Africa [3-5]. The global burden due to schistosomiasis is currently estimated at 1.7-4.5 million disability-adjusted life years (DALYs) [2,6,7]. Schistosomiasis predominantly occurs in tropical and sub-tropical areas, but the disease is also seen in specialized travel clinics in Europe and North America among migrants and returning travellers [8,9]. Schistosomiasis can cause acute illness, but its main public health impact is due to chronic infection, including increased risk for anaemia, growth stunting and under-nutrition in affected populations, as well as exacerbation of co-infections and impairment of cognitive development and work capacity [10]. Depending on the species of *Schistosoma*, the liver, intestine, spleen, lungs and urogenital system are affected, which can cause serious health problems in later life [10]. For example, chronic infection with *Schistosoma haematobium* is associated with bladder cancer, and might account for up to 30% of all cancer cases in some endemic regions [11,12].

Humans become infected with *S. haematobium* when they wade, swim or bathe in water, inhabited by compatible intermediate snail hosts, previously contaminated by human urine containing parasite eggs. The adult schistosomes live as paired male and female worms in the perivesical venous plexus of their human host. Eggs produced by female worms get trapped in the tissues and can lead to inflammatory and obstructive disease in the urogenital system [3]. Eggs that pass through the bladder wall are voided in urine. If urination occurs in freshwater, the parasite eggs will hatch in the water and release miracidia (a free swimming larval stage), which subsequently infect the specific intermediate host snails. In Zanzibar, the only intermediate host snail for *S. haematobium* is *Bulinus globosus*[13]. In the snail, asexual reproduction takes place and 4–6 weeks after infection, the snail starts to shed cercariae, the human infective larval stage, into the water. Importantly, a single snail can shed thousands of cercariae over its life-time. On locating a human host, cercariae penetrate the skin and, after migrating via the lungs to the liver develop, within 9–10 weeks, into adult separate sex worms. These worms pair up, and on average, live and produce eggs for 3–5 years. Many eggs leave the body via the excreta, but many more become trapped in the tissues causing an inflammatory reaction that leads to morbidity [3,14,15].

In Zanzibar, the population is at risk of infection with only *S. haematobium*[13,16], the schistosome species that affects the bladder, genital tract and urethras [17-20], causing urogenital schistosomiasis. The highest prevalence of *S. haematobium* infection is found among school-aged children on both islands, Unguja and Pemba [21-25]. Previous research has shown that visible haematuria, microhaematuria and urinary and genital tract pathology are associated with infection, particularly heavy intensity infections [24-27].

### Control of schistosomiasis with focus on efforts in Zanzibar

To interrupt the life cycle of *S. haematobium*, there are four main strategies: (i) kill the worms in humans, by anthelminthic drugs; (ii) kill the intermediate host snails, including those carrying intramolluscan parasites, by chemical (i.e. molluscicides) or biological control agents (e.g. competitor snails and fish); (iii) stop people infecting snails, by convincing them not to urinate into open freshwater bodies; and (iv) stop cercariae infecting man, by keeping people out of infested water bodies [28,29].

The current global strategy to control morbidity due to schistosomiasis is preventive chemotherapy that is the regular administration of the anthelminthic drug praziquantel to at-risk populations (e.g. school-aged children) without prior diagnosis [30]. In the frame of preventive chemotherapy programmes in Zanzibar, praziquantel has been administered to school-aged children on both islands since 1994 by multiple rounds of treatment depending on the availability of external funds [23,24,31-33]. As a result, the prevalence and intensity of *S. haematobium* infection in school-aged children in Unguja and Pemba decreased considerably. For example, in Unguja, while a prevalence of >50% was recorded in the early 1980s, it has dropped to <10% in 2006 [23,34,35]. Hence, morbidity control has largely been achieved in Zanzibar. Of note, in a survey carried out in March 2011 in 24 schools on each island, the overall prevalence of *S. haematobium* was 8% in Unguja and 15% in Pemba.

### Current initiatives for schistosomiasis control in Zanzibar

In mid-2010, the Zanzibar government expressed commitment to eliminate urogenital schistosomiasis on the islands of Unguja and Pemba. As backbone for this commitment the Zanzibar Neglected Tropical Disease (NTD) Programme will be implementing its “3-year comprehensive strategic plan to combat neglected tropical diseases in Zanzibar 2009/2011” [36], referred to as the “National Plan”. It calls for preventive chemotherapy using praziquantel, accompanied by health education and community mobilization activities to consolidate and enhance the impact of preventive chemotherapy. The National Plan is focusing on the districts, and zones within districts, where urogenital schistosomiasis is known to be endemic.

In support of the National Plan, the World Health Organization (WHO) has agreed to supply sufficient quantities of praziquantel to ensure repeated rounds of preventive chemotherapy and the Schistosomiasis Control Initiative (SCI) will assist with the drug intervention at a large scale. WHO and SCI have long histories of working with the Zanzibar Ministry of Health (MoH) in their fight against schistosomiasis and soil-transmitted helminthiasis [18,37].

An international consortium, called Zanzibar Elimination of Schistosomiasis Transmission (ZEST), is committed to assisting the government of Zanzibar in its efforts to eliminate urogenital schistosomiasis. This consortium includes the Zanzibar MoH, including the Zanzibar NTD Control Programme, the Public Health Laboratory - Ivo de Carneri (PHL-IdC) Pemba, Zanzibar government agencies, WHO, SCI, the Natural History Museum (NHM) in London, the Swiss Tropical and Public Health Institute (Swiss TPH) in Basel, the London School of Hygiene and Tropical Medicine (LSHTM), the University of New Mexico and the Schistosomiasis Consortium for Operational Research and Evaluation (SCORE) based at the University of Georgia, with additional groups expected to join as the efforts progress. Within the ZEST project, the emphasis will shift from morbidity control to comparative methods of transmission control and local elimination of urogenital schistosomiasis. In addition to measuring the outcomes of this project, thorough documentation of the process, including lessons learned, will be crucial so that schistosomiasis control and elimination programmes implemented elsewhere can benefit from the ZEST experiences.

### Research for schistosomiasis elimination in Zanzibar

SCORE was established in December 2008 to address operational research questions pertaining to gaining and sustaining control of schistosomiasis, and to establish the proof-of-concept that local elimination is feasible. SCORE is funded through a 5-year grant by the Bill & Melinda Gates Foundation awarded to the University of Georgia Research Foundation (UGARF). The goal of SCORE is to provide pragmatic answers that will help current and future schistosomiasis control programme managers to more efficiently control the disease more efficiently. This includes learning which approaches need to be pursued for controlling and eliminating schistosomiasis, as well as developing and validating new tools and strategies. SCORE’s vision is that the work will inform efforts to gain control of schistosomiasis in high-prevalence areas, sustain control and move towards elimination in areas of moderate prevalence, and ultimately eliminate schistosomiasis in areas of low endemicity.

Increased use of praziquantel through preventive chemotherapy and availability of treatments for those in need will be central to the success of the National Plan to control and eliminate schistosomiasis. At the same time, increased efforts are required to reduce transmission of the disease. Layered onto the implementation of the National Plan by the Zanzibar government, SCORE, under the umbrella of the ZEST partners, will simultaneously study and implement additional measures of schistosomiasis control/elimination.

Three promising interventions were discussed at ZEST meetings in Zanzibar in 2011 as possible add-ons to preventive chemotherapy to be implemented as part of the National Plan. These are: (i) water and sanitation changes; (ii) snail control; and (iii) human behaviour change interventions that could interrupt the transmission cycle of *S. haematobium*. The Zanzibar Water Authority (ZAWA) has funding to provide piped water and potentially improved sanitation, but such interventions will take considerable time and effort and are unlikely to make widespread progress within the timeframe of ZEST. Therefore, the SCORE study presented here will focus on evaluating the impact of snail control using the molluscicide niclosamide and environmental management and on a community-informed behaviour change intervention on rates and success of efforts to eliminate schistosomiasis in Zanzibar, in parallel with the National Plan.

### Goal, aims and objectives

The goal of this project is to provide an evidence-base for programme decisions about schistosomiasis elimination, not only for the Zanzibar NTD Control Programme, but also for other settings in Africa and elsewhere that aim to eliminate schistosomiasis. The study will be implemented on Unguja and Pemba islands and will compare snail control and behaviour change strategies as complementary measures to preventive chemotherapy in three study arms, each consisting of 15 communities (shehias). Hence the study involves a total of 45 study communities on each island. The following aims and specific objectives are related to this goal.

#### Aims

1. Eliminate schistosomiasis as a public health problem* on Unguja in 3 years and interrupt transmission in 5 years.

2. Control schistosomiasis throughout Pemba (prevalence <10% in school-aged children) in 3 years and eliminate it as a public health problem* in 5 years.

3. To identify effective behaviour change strategies with an understanding of the associated costs, motivators, triggers and barriers associated with behaviour change interventions.

4. To identify effective snail control strategies with an understanding of the associated costs, motivators, triggers and barriers associated with snail control interventions.

* Eliminate schistosomiasis as a public health problem is defined: "Reduction of *Schistosoma* prevalence to <1% heavy infections based upon direct egg-detection methods in the school-aged population; continued intervention measures are required to prevent resurgence of transmission" as in Rollinson et al. 2012 [29].

#### Specific objectives, activities and milestones

1. To assess annually the reduction in prevalence and intensity of *S. haematobium* infection according to standardized, quality-controlled methods (i.e. urine filtration and reagent strip testing) in schoolchildren aged 9–12 years within and between each study arm from year 1 to year 5.

2. To assess the reduction in the prevalence and intensity of *S. haematobium* infection as measured by urine filtration and reagent strip testing in adults in year 1 and in year 5 within and between each study arm.

3. To assess the reduction in the prevalence and intensity of *S. haematobium* infection as measured by urine filtration and reagent strip testing in first-year schoolchildren in year 1 and in year 5 within and between each study arm.

4. To assess the difference in the sero-prevalence of *S. haematobium* infection in first-year schoolchildren after 4 years of control within and between each study arm.

5. To assess annually changes in knowledge on *S. haematobium* transmission and the impact of change of behaviour on transmission.

6. To assess the impact of niclosamide on the presence of *B. globosus* in freshwater ponds and rivers.

7. To test and evaluate recently developed methods for the diagnosis of *S. haematobium* infections (such as: enzyme linked immuno-sorbent assay (ELISA) for assessment of antibody levels in blood; polymerase chain reaction (PCR) for DNA detection in urine) for their sensitivity, specificity and feasibility for elimination programmes.

8. To create, test and validate mathematical models for the prediction of *S. haematobium* prevalence after control interventions (to be developed with external partners).

## Methods/Design

### Study area and population

The zanzibar archipelago is part of the United Republic of Tanzania and consists of the two main islands, Unguja and Pemba [38], as well as a few much smaller islands. There are two annual wet seasons: the Masika rains from the south lasting usually from March to June, and the Vuli rains from north-east occurring from October to November. The average annual temperature ranges between 23°C and 32°C. The estimated total resident population for Unguja and Pemba was 773,234 and 511,576 inhabitants, respectively, in 2010 [39]. Islam is the predominant religion [40]. The main economic activities include seawater fishing and cash crop production (e.g. coconuts, cloves, chillies, copra and seaweed). Tourism is of growing importance, since the islands offer a host of historical and natural attractions [39].

Both islands are divided into large administrative areas (i.e. districts). The districts are sub-divided into smaller administrative units (i.e. shehias). Unguja consists of six districts (North A, North B, Central, West, South and Urban) and a total of 203 shehias. Pemba consists of four districts (Micheweni, Wete, Chake and Mkoani), subdivided into 88 shehias. In Unguja, schistosomiasis is endemic in districts North A, North B, Central, West and a part of the Urban district (entitled “Zone C” for the operational ease of preventive chemotherapy distribution), whereas all four districts of Pemba are endemic. The schistosome-endemic areas will be targeted for preventive chemotherapy within the National Plan.

### Interventions in the frame of SCORE and the National Plan

**Figure 1:**
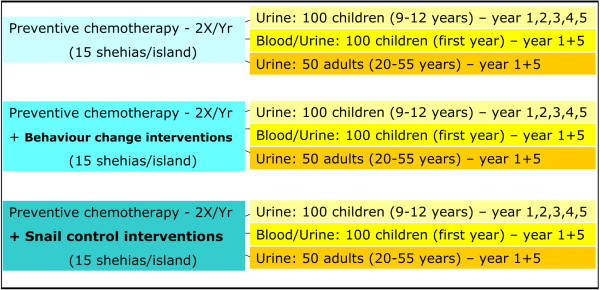
**Study arms to assess the impact of different *S. haematobium *control interventions in Zanzibar.** In a randomized intervention trial with three study arms we will compare the impact of (i) praziquantel administration as implemented according to the National Plan of the Zanzibar government, (ii) behaviour change interventions in addition to praziquantel administration and (iii) snail control in addition to praziquantel administration on *S. haematobium* infection prevalences and intensities in Zanzibar (Unguja and Pemba islands). Each study arm comprises 15 communities; hence, a total of 45 communities per island is surveyed. The number of study participants per study arm and community is given in Figure 1.

The SCORE research study is designed as a randomized trial to be layered on the planned preventive chemotherapy campaigns to be conducted by the Zanzibar MoH. The trial will have three study arms, each comprising 15 shehias. Hence, on each island, a total of 45 shehias will be included into the study. The three study arms are designed as follows (Figure 1):

i) Implementation of the National Plan of the Zanzibar government (preventive chemotherapy, health education and community mobilization focused on preventive chemotherapy).

ii) Implementation of the National Plan plus targeted snail control.

iii) Implementation of the National Plan plus an intensive behavioural intervention aimed at reducing *S. haematobium* transmission.

#### Preventive chemotherapy in the frame of the National Plan

In the frame of the National Plan of the Zanzibar MoH, preventive chemotherapy with praziquantel (40 mg/kg using a dose pole) [41] against schistosomiasis and albendazole (400 mg) against soil-transmitted helminthiasis will be conducted twice each year, at 6-month intervals to all children aged >2 years and all adults throughout the islands. Severely sick people and pregnant women will be excluded from preventive chemotherapy interventions. However, presumptive treatment of pregnant women at any stage of pregnancy will be implemented at mother and child health (MCH) facilities, once in pregnancy, in all the areas where schistosomiasis is transmitted.

The anthelminthic drugs for the preventive chemotherapy campaigns will be donated by WHO, whereas SCI will financially support the implementation of drug delivery. In addition to preventive chemotherapy, the National Plan will promote health education and community mobilization campaigns.

#### Snail control

*B. globosus* is the only intermediate host snail for *S. haematobium* in Zanzibar and occurs commonly in a variety of temporary and permanent habitats on Pemba and Unguja [13]. Snail populations can be reduced either indirectly by the destruction of their natural habitat, for example by the removal of vegetation and debris from riverbanks (environmental management), or directly by the use of a molluscicide (chemical control). Environmental management and mollusciciding represent important, well-tried and effective tools to supplement preventive chemotherapy against schistosomiasis [29,42,43]. The molluscicide niclosamide has been recommended by WHO to control human schistosomiasis [44-46]. It has been applied earlier on Unguja in a study for the control of schistosomiasis in the early 1980s [35], and has been widely and effectively used for snail control in schistosomiasis control programmes in Morocco, Egypt and the People’s Republic of China [47-50]. Moreover, niclosamide 70% wettable powder (WP) was tested in a pilot study in Zanzibar in June-August 2011, preceding the study proposed here.

In the randomly selected shehias of the current study, niclosamide 70% WP will be applied to open water bodies (slow flowing rivers, ponds and lakes) in concordance with preventive chemotherapy. Since, the optimum time for administering praziquantel is when the snail populations are absent and there is no risk of re-infection for the treated population, mollusciciding is most effective if applied immediately before a pre-planned chemotherapy campaign to avoid immediate re-infection [28]. Areas where humans are in regular contact with open freshwater bodies (e.g. for washing or bathing) in the selected shehias will be identified and treated with niclosamide 70% WP in the months preceding preventive chemotherapy in Zanzibar, aiming for two applications per year. Before each intervention, all involved shehas (community leaders) and community members will be informed about the purpose of the studies and their concerns and suggestions will be taken into consideration. Since snails often lay eggs on rubbish in the rivers and ponds, the community members will be encouraged to engage in the clearance of vegetation and rubbish from open water bodies; especially from potential transmission sites where humans have regular water contact. Full safety briefings will be required.

#### Behaviour change interventions

School-aged children who live in *S. haematobium*-endemic areas are usually at the highest risk of becoming infected and are most likely to be involved in transmitting the disease, because they tend to spend time swimming or playing in open water bodies that may contain infected snails and because children have no or limited protective immunity [51-54]. Adults are also at risk of infection through bathing or washing clothes in contaminated water or through occupational exposures (e.g. fishing, rice farming and car washing) [40,55]. Health communication taking into account local knowledge, attitudes and practices and the integration of communities in priority settings, decision making and planning of schistosomiasis control interventions, will be essential to achieve a change in behaviour [56].

The behaviours we believe most important to modify in order to reduce schistosomiasis transmission in Zanzibar are (i) children urinating in streams and ponds and (ii) children playing, swimming and washing laundry in the same streams and ponds. The urination behaviour is particularly difficult to target because it is not observable, and children with schistosomiasis may have increased urgency and difficulty in restraining urination because of bladder irritation from the parasite. Additionally, with little else to do, rivers and ponds are a source of recreation for children.

The behavioural intervention is being guided by human-centred design (HCD) processes and techniques [57]. This process starts with a specific challenge such as the prevention of children urinating in rivers and ponds. In partnership with the community we explore community knowledge, beliefs, current practices and social norms related to the challenge through formative qualitative methods. The resulting findings are shared with the community and together through a participatory process a behavioural intervention is designed and implemented as concrete and tangible solutions. This process helped to identify cultural norms around urination in freshwater bodies and messages, activities and approaches that might be useful to change children’s behaviour in Zanzibar. In addition we identified alternative structural solutions and replacement behaviours to urinating in the water that might be acceptable in Zanzibar, as well as best channels and approaches for communications [58]. Behavioural interventions based on formative findings and the participatory work of the community are being designed to raise awareness of schistosomiasis transmission, diagnosis, treatment and prevention and to influence the adoption of new behaviours by: (i) training teachers, coaches and students in local primary schools; (ii) training teachers and students in religious schools; (iii) designing and installing locally produced male and female urinals at targeted water hotspots where children congregate; (iv) providing alternative play activities and play structures for children; and (v) providing washing platforms at designated tap water sources and areas a short distance from local washing water sources.

### Justification of the number of participants

With regard to the goal and aims of the SCORE study, the comparison of outcomes between the three intervention arms will document benefits of interventions added to preventive chemotherapy. However, due to the overall low *S. haematobium* prevalence in Unguja (8% in school-aged children, as determined in 24 schools surveyed in March 2011) and the estimated further decrease in prevalence due to biannual treatment in the coming 3 years, the difference attributed to additional interventions could be very small. Hence, to reach a desired power of 80% in our randomized trial, we would need a sample size of clusters (i.e. shehias) that exceeds the total number of shehias in schistosomiasis-endemic settings in Unguja and Pemba and a sample size of participants that is not logistically feasible. The choice of 15 shehias per intervention arm per island, and the number of people to be tested (per island: 4,500 children aged 9–12 years annually, 4,500 first-year schoolchildren in years 1 and 5 and 2,250 adults in years 1 and 5) is a compromise between what is optimal and what is practically achievable.

### Selection and randomization of study shehias and participants

#### Eligibility and randomization of shehias

Unguja and Pemba are divided into 203 and 88 shehias, respectively. The following exclusion criteria were applied. First, in Unguja, all shehias with no endemic schistosomiasis according to expert opinion (n=104) and in Pemba all shehias without a stream indicated on an aerial photography (n=13) were excluded. Second, all shehias without a primary school were excluded (Unguja: n=43; Pemba: n=23). Third, if a shehia had more than one primary school, the school with lower pupil numbers was excluded. Fourth, all shehias with schools that were attended by fewer than 200 children in 2008 (most recent available data) were excluded (Pemba: n=1). Primary schools in Zanzibar include school grades 1–7, consisting of children mainly aged between 7 and 13 years. Hence, we anticipate that, in a school attended by at least 200 children in 2008, there are at least 100 children aged 9–12 years that can be enrolled for the current study. Fifth, one shehia in Unguja and one shehia in Pemba were excluded as the indicated school was not located in the same shehia. Adhering to these inclusion and exclusion criteria resulted in 45 eligible shehias in Unguja and 50 eligible shehias in Pemba.

**Figure 2:**
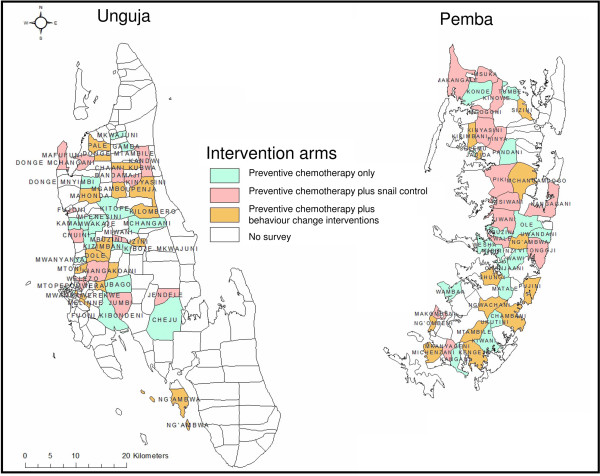
**Assignment of communities (shehias) to control interventions aiming to eliminate urogenital schistosomiasis in Zanzibar.** On both, Unguja and Pemba islands, 45 shehias were randomly assigned to one of three intervention arms: 15 shehias on each island will receive praziquantel administration as implemented according to the National Plan of the Zanzibar government, 15 shehias will be targeted by behaviour change interventions in addition to praziquantel administration and 15 shehias will receive snail control in addition to praziquantel administration.

For the random selection and allocation of 45 shehias both in Unguja and Pemba to one of the three intervention arms, in a first step, all eligible shehias having participated in the annual 24-school surveys formerly conducted by the *Piga vita Kichocho* programme [23] (Unguja: n=13; Pemba: n=17) were included in a computer-based randomization to one of the three study arms. In a second step, out of the remaining 33 shehias in Pemba, 28 were randomly selected to be part of the study. In a third step, the 32 shehias in Unguja and the 28 shehias in Pemba were randomized to one of the three study arms. Although the randomization may have resulted in differences in the starting prevalence of *S. haematobium* infection or other factors among study arms, we did not re-randomize. Figure 2 shows the distribution of shehias to each of the three study arms in Unguja and Pemba.

#### Eligibility and randomization of schoolchildren

In years 1 to 5 of the study, all schoolchildren aged 9–12 years and in years 1 and 5 additionally all first-year schoolchildren are eligible to participate in the study. For the respective surveys, (i) 130 children aged 9–12 years and (ii) 130 first-year schoolchildren will be randomly selected for urine collection and additional finger-prick blood collection for first-year schoolchildren. For this purpose, all eligible children will line up, in separate lines for boys and girls and school class. Subsequently, we will systematically select each third child in the lines to be included in the study. This procedure will be continued until we reach a total of 130 selected children, accounting for a 20% drop-out with the final aim to sample 100 children aged 9–12 years and 100 first-year schoolchildren. We anticipate to collect each year 9,000 urine samples from 9-12-year-old children and 9,000 urine and finger-prick blood samples from first-year schoolchildren in years 1 and 5 on each of the two islands.

#### Eligibility and randomization of adults from communities

All adults aged 20–55 years are eligible to participate in the study. In years 1 and 5, we will select a quota sample of 50 houses per shehia according to a method suggested by Winkler et al. [59]. For this purpose, a gyro with a marked arrow-star (equal to the number of field interviewers in a team) pointing into different directions will be spun on a central point in the shehia. Subsequently, each interviewer will count the households to the edge of the shehia following a path in direction of the arrow. On return to the centre point, the number of households in that direction will be reported and the interviewer will state a number within the range of counted households. This number will be compared to a list with computer-generated random numbers created for each shehia. The random number corresponding to the number stated by the interviewer will assign where to start the first questionnaire interview. Proximity sampling will then be pursued with interviewers moving from one household to the next nearest household until 50 households are covered. People sharing the same kitchen or pot will define a household. If more than one of the present household members is eligible to participate, we will use a “drawing cards” approach for randomly selecting only one of the members to participate in the study. We plan to collect 4,500 urine samples and questionnaire data from adults aged 20–55 years in years 1 and 5 on each of the two islands.

### Data collection

#### Recruitment of participants and collection of specimen and questionnaire data

The community leaders and headmasters of the selected shehias and primary schools will be informed about the aims and procedures of the study. Each year, in the selected schools, the teachers will be informed about the forthcoming activities. The study will be explained in lay terms to the children and they will be asked for their oral assent to participate. Children will be selected according to the eligibility criteria and randomization as described above. Name, sex, age, school grade and village and shehia of residency of the selected children will be recorded and the children will be provided with an information and consent form, the latter to be signed by their parents or legal guardians and to be returned the following day. On that day, children who submitted a signed consent form will be provided with a plastic container (120 ml) labelled with the individual’s ID, for subsequent urine collection on the spot between 10 AM and 12 AM. From the selected children from the first-year at school, in addition to a urine sample, a finger-prick blood sample will be collected in small tubes (BD Microtainer, Ref.: 365967; BD, Oxford, UK) labelled with the individual’s ID and stored on ice after clotting.

In the study communities, the shehas will be informed about the purpose and procedures of the study and invited to inform the community about the forthcoming visits of households. The sheha will be interviewed in Kiswahili with a “village inventory form” and asked a set of questions on the demographic characteristics of his shehia. In addition to general information, data on occupation of the population closely related to water contact, on health facilities and available drugs, on water contact sites, on availability and use of water sources and sanitary facilities will be collected.

While visiting the households selected as described above, the study procedure will be explained to the present household members in Kiswahili and they will be asked for their oral assent to participate. The selected household member will be invited to sign a written informed consent form, given a urine collection container labelled with a unique ID and asked to provide a urine sample on the spot, ideally produced between 10 AM and 2 PM of the same day. Additionally, the participant’s ID, sex, age, occupation, place of birth, time of residency in the respective shehia and sanitary behaviour will be recorded using a pre-tested questionnaire administered in Kiswahili.

### *Laboratory procedures to assess* S. haematobium *infection in humans*

In the laboratory, each urine sample will be screened for visual blood (macrohaematuria) and for microhaematuria using reagent strips (Haemastix; Siemens Healthcare Diagnostics GmbH, Eschborn, Germany). When tested visually, the colour of the urine will be coded semi-quantitatively according to a pretested colour chart (1, 2, 3, 4, 5, 6); the colorimetric test of the reagent strip for microhaematuria will be recorded semi-quantitatively (0 = negative, 1 = +, 2 = ++, 3 = +++, 4 = trace). Additionally, urine samples will be vigorously shaken and 10 ml of each sample will be filtered using a plastic syringe with a filter-holder containing a 13 mm polycarbonate filter (Sterlitech, Kent, WA, USA) [60]. All *S. haematobium* eggs present on the filter will be counted under a microscope by experienced laboratory technicians and exact egg counts will be recorded for each individual.

From a subset of fresh urine samples with microhaematuria according to positive reagent strip tests, *S. haematobium* eggs will be hatched and miracidia collected and stored on Whatman FTA indicator cards (Whatman plc, Maidstone, UK) [61] for population genetic studies layered onto the current study to evaluate the impact of the interventions on the genetic diversity and population structure of the schistosomes in the shehias.

Additionally, on each survey day in years 1 and 5, from every 10^th^ of the collected urines of sufficient amount after the urine filtration has been performed, 10 ml will be transferred into 15 ml Falcon tubes, labelled with the ID of the respective participant and stored for future use at −20°C at HCLU/PHL-IdC. The frozen samples will be examined for *S. haematobium* infections using novel sub-microscopic diagnostic approaches (i.e. PCR or ELISA) evaluated either within SCORE studies or by external partners who have yet to be identified.

The clotted finger-prick blood samples will be centrifuged at 6,000 *g* for 10 min immediately after arrival at HCLU/PHL-IdC. The sera will be transferred into semi-skirted 96 well plates (maximum 250 μl), labelled and stored at −80°C at PHL-IdC pending further analyses. Antibody levels against *S. haematobium* antigen will be tested using ELISA, following the manufacturer’s instructions or using newly developed multiplexing assays.

For quality control, microscope slides with the filter containing potential *S. haematobium* eggs will be covered with cellophane soaked in glycerol and stored in slide storing boxes that are kept at room temperature. After each survey, 10% of the slides of each technician will be re-read by external senior laboratory technicians by adding a drop of Lugol’s solution on the hydrophilic cellophane. The number of *S. haematobium* eggs will be recorded and compared with the original egg counts. In the case of significant discrepancies between the original and re-read slides (false negatives, false positives, egg counts resulting in a different infection intensity category) that exceed a defined threshold, all stored slides of the respective technician will be re-examined.

#### Monitoring of snail populations and infection level

Preceding each round of preventive chemotherapy, transmission sites at open water bodies where humans have regular contact with water (identified on maps and by consulting community members and children) in the 15 selected shehias on both islands will be treated with niclosamide 70% WP. The number of freshwater bodies treated, their kind, size and exact location will be recorded. The amount of niclosamide 70% WP used will be recorded after each intervention. Before treating the water bodies, snail densities will be assessed at each site. For this purpose, a sample area of 15 m^2^ will be measured and surveyed for any snails for 15 min by two trained staff. All snails will be deposited into a basin and all organisms alive and dead in the collection tube will be counted and recorded. Snails will be classified to species level. Once the number and species of snails and the genus of the other organisms have been recorded, all organisms, except *B. globosus*, will be returned and evenly distributed to their original collection sites. Temperature, pH, salinity and conductivity of the water will be measured and recorded at each of the sites, on all survey and sample days using standard protocols and forms. All collection sites will be located using a hand-held global positioning system (GPS) device (Garmin GPSMap 62, Garmin (Europe) Ltd; Southhampton, UK).

*B. globosus* snails will be transferred to HCLU in Unguja and PHL-IdC in Pemba to determine whether they are infected with *S. haematobium*. Snails will be investigated for parasitic infection using the shedding method. For this purpose the snails will placed individually in flat-bottomed glass vials (height: 7.5 cm, diameter: 2.5 cm) containing dechlorinated water, and exposed to indirect sunlight for a maximum duration of 4 hours [62]. Cercariae shedding will be observed using a dissection microscope. Snails that do not shed cercariae on the first sunlight exposure will be re-exposed on the second day. Based on their morphology, cercariae will be categorized either as those of *S. haematobium* or those of other trematodes (non-*S. haematobium* cercariae) [63]. *S. haematobium* cercariae will be collected using a pipette and placed on Whatman FTA cards for future molecular analysis. All collected snails will be preserved in small glass tubes containing 100% ethanol, which will be deposited in the schistosomiasis repository (SCAN; http://www.nhm.ac.uk/research-curation/collections/curation-groups/scan/index.html) held at NHM in London, and made available for future investigations as needed.

#### Assessment of behaviour change

The impact of interventions on the behaviour of people living in the targeted 15 shehias in the behaviour change study arm on each island will be assessed in qualitative baseline and annual follow-up studies. In-depth interviews (IDIs) and focus group discussions (FGDs) with young and older schoolchildren, teachers, parents and community leaders will be conducted in Kiswahili by trained members of the Zanzibar NTD Programme. The content of each FGD and IDI will be recorded and transcribed into English and analyzed through a modified grounded theory approach. Additionally, pre-tested structured observation checklists will be used to capture critical observations of the same behaviours in households and public venues. Brief questionnaires, based upon behavioural theory constructs, will be administered to measure behavioural determinants associated with the intervention implementation process and to assess its impact on *S. haematobium* infections in schoolchildren and communities.

#### Collection of additional data relevant for the study outcomes

Baseline data on the 90 selected shehias will be collected from the existing health management information system (HMIS), the Office of the Chief Government Statistician Zanzibar, available aerial photographs, and by consulting the local authorities (shehas and school headmasters) and key informants. This baseline information will include data on the area of the shehias (in km^2^), number of inhabitants, number and size of primary and secondary schools, number of public and private health centres, number of MCH facilities, water sources for private and domestic use (e.g. piped water, wells, rivers and ponds), sanitation coverage (e.g. number of pit latrines, ventilated improved pit latrines, flush toilets), immigration and main income source of the population, mainly covered within the village inventory form. To track multiple factors that could affect study outcomes, shehas will be interviewed on an annual basis. In addition to direct village inventories, we will gather and store reports from non-governmental organisations (NGOs) involved in water and sanitation as well as other health-related projects and be in regular contact and exchange with ZAWA to track their activities.

Praziquantel treatments in community health centres will be recorded and the data transferred to the HMIS of the MoH. In relation to preventive chemotherapy administration according to the National Plan, the Zanzibar MoH will collect data on treatment coverage, use of praziquantel from primary health care units (PHCUs) and treatment of pregnant women in MCH facilities. Costs incurred for all activities will be recorded and used to estimate the cost-effectiveness of the different intervention arms.

#### Data management and analysis

Quantitative data from laboratory examinations, questionnaires and snail records will be entered in Microsoft Excel version 10.0 (2002 Microsoft Corporation) or EpiInfo version 3.5.1 (Centers for Disease Control and Prevention; Atlanta, GA, USA) by local staff in Zanzibar. Statistical analyses will be carried out with STATA version 10 (StataCorp.; College Station, TX, USA). Descriptive and regression analyses will be conducted to evaluate the effectiveness of the interventions under study, including evaluation of cost and cost-effectiveness. In addition, spatial analysis will be conducted to identify clusters and spatially defined factors that may be modifying outcomes. For each year, *S. haematobium* prevalence and infection intensity data will be calculated. The results from the different study arms will be compared on an annual basis, after 2 years of intervention and at the end of the 4-year intervention period.

For the assessment of effectiveness of behaviour change interventions, FGDs and IDIs will be transcribed verbatim, translated into English by bilingual research assistants and entered as Microsoft Word documents into Atlas-ti version 6.0 (Software Development GmbH; Berlin, Germany) to facilitate text searching, data coding and analysis. Open, axial and selective coding will be used to analyse the transcribed narratives. Open coding and a word-by-word analysis will be used to identify, name and categorize explanations and descriptions of the day-to-day reality of participants as related to schistosomiasis and other water-related issues. Structured observations will be summarised and descriptive open-ended questions analysed in the same manner for the FGDs and IDIs narratives.

#### Data storage and handling

All original data records will remain within the office of the HCLU or PHL-IdC of the MoH Zanzibar. Electronic data files will be transferred to the NHM in London and Swiss TPH in Basel and original and cleaned data will be stored in a password-protected folder accessible for all investigators. Name-linked information on participants will remain confidential and be shared only by the study team. Unique identifiers will be used for data records, transcripts and computer-based software data management. When discussing or showing the results of analyses in public venues, the information will always be reported at an aggregate level so that individual participants cannot be identified.

### Protocol review and ethical clearance

The study protocol summarised here was reviewed by the SCORE secretariat consisting of several scientists with long-term experience in the epidemiology and control of schistosomiasis before it was submitted to and accepted by (i) the Zanzibar Medical Research Ethical Committee (ZAMREC), (ii) the “Ethikkomission beider Basel” (EKBB) in Switzerland, and (iii) the institutional review board of the University of Georgia (IRB UGA). The trial has been registered at the International Standard Randomised Controlled Trial Number Register (ISRCTN48837681; http://www.controlled-trials.com/ISRCTN48837681).

At the onset of each study segment, all participants will be informed about the purpose and procedures of the study and the respective study segment and asked to submit a written informed consent prior to collection of urine or finger-prick blood samples, or the collection of questionnaire, FGD or IDI data. The obtained information will be treated with strict confidentiality and data made anonymous before analysis.

## Discussion

In November 2011, the Secretariat of the WHO considered that elimination of schistosomiasis “is feasible in all epidemiological settings, provided that there is strong political commitment to the goal, supplies of anthelminthic medicines for preventive chemotherapy are adequate, and support is provided by the international community” [64]. Further, the Executive Board called “on all countries endemic for schistosomiasis to intensify control interventions and strengthen surveillance, with the aim of eliminating the disease” [64]. A few months later, in May 2012, the 65^th^ World Health Assembly announced that schistosomiasis elimination campaigns should be initiated where appropriate [65].

The islands of Zanzibar are meeting the requirements specified by the secretariat, rendering the archipelago a suitable candidate for schistosomiasis elimination. Control of schistosomiasis has a long-term history on Unguja and Pemba [18,19,23,35,66,67]. Elimination of urogenital schistosomiasis on the islands of Zanzibar is a health priority, endorsed by the President, and the Zanzibar MoH is committed to fulfil the National Plan [36]. The MoH and the Zanzibar NTD Programme have backing from important and influential partners such as WHO and SCI, which will strongly support the control and elimination efforts on both islands by donating praziquantel and funding treatment implementation costs, respectively. The rigid boundaries of the islands, the strong public health system and a number of successful previous campaigns against malaria [68], cholera [38], lymphatic filariasis [69], soil-transmitted helminthiasis [70,71] and schistosomiasis [18,37] have sensitized the population [72]. Huge steps towards elimination of lymphatic filariasis and malaria in Zanzibar have been made [68,69] and the elimination of urogenital schistosomiasis in Unguja seems now an achievable goal [29,73].

However, a broad consensus has been reached in the international community that long-term commitment by influential partners and efforts going beyond preventive chemotherapy are needed to achieve elimination [29,74,75]. Indeed, examples of countries having achieved schistosomiasis elimination show that preventive chemotherapy must be fully integrated with other tools of transmission control such as mollusciciding against snail intermediate hosts, changing behaviour to avoid the contamination of water bodies and prevent (re-)infection and improving water and sanitation [29,75-77].

The study on urogenital schistosomiasis elimination in Zanzibar that we describe here will contribute to identifying what, in addition to preventive chemotherapy, needs to be done to prevent, control and ultimately eliminate schistosomiasis, and to draw lessons for current and future schistosomiasis elimination programmes. It will provide important information for the Zanzibar NTD Programme and for other endemic areas in Africa and elsewhere that aim to eliminate schistosomiasis, and particularly urogenital schistosomiasis.

Based on the findings and outcomes of the proposed study, and in close collaboration with WHO and other partners, new guidelines for schistosomiasis control programmes progressing from morbidity control to transmission control/local elimination may be developed.

## Competing interests

The authors declare that they have no competing interests.

## Authors’ contributions

SK drafted the original protocol and manuscript. KAM, SaMA and DR initiated the study. ISK and ShMA advised on local conditions and distinctions. SK, ISK, ShMA and JU developed the parasitological part. SK, ISK, ShMA and DR focussed on the snail control design. BP, KAM and SaMA developed the behaviour change component of the study. SK, KAM, SaMA, ISK, ShMA, MA, AG, JU and DR are substantially involved in the study preparation and conduction. MA, AG, AF, LS, DGC, JU, KAM and DR provided expert knowledge for the study design and implementation and support the study on all levels. All authors contributed to the full conception and design of the study, revised the manuscript and approved its final version.

## Pre-publication history

The pre-publication history for this paper can be accessed here:

http://www.biomedcentral.com/1471-2458/12/930/prepub
